# Nurses Unemployed and Underpaid

**Published:** 1919-11-01

**Authors:** 


*To the Editor of* THE HOSPITAL.

### NURSES UNEMPLOYED AND UNDERPAID.

*To the Editor of* THE HOSPITAL.

SIR,—In your issue dated October 18, 1919, I observe a spirited protest by Miss Seymour Yapp *re* the inadequate salary offered to the matron of Birkenhead Infirmary by the Guardians.

Every trained nurse must agree with Miss Yapp that the salary offered is too small, but every nurse who at the present moment is out of employment (and every matron, too) will ponder over her statement that "every trained nurse who takes an underpaid appointment makes it harder for those who come after." Miss Yapp does not realise that trained nurses now out of employment are thankful to take any post rather than face the coming winter with its privations. Three weeks ago there were 130 matrons out of employment. One hundred pounds a year could not be thrown lightly away because of those who "came after." Whilst so many qualified women are at a loss for employment (through having offered themselves for war service), these under-paid appointments will be thankfully taken as an alternative to starvation. I myself have been unemployed for months rather than take what I consider an inadequate salary in return for my experience. The result is now that I am obliged to take a post yielding me £50 a year—a less sum than my staff nurse received in the hospital of which I was matron for four years. I am general, fever, and mental trained, and have had a great deal of children's experience, in addition to catering, secretarial, and hospital administrative work. I assert that owing to the large number of unemployed nurses, employers are deliberately offering as little as they can, as the supply exceeds the demand. The College is doing fine work for the nurses, and I believe they refuse to insert employers' names in their unemployment bureau who will not promise a fair salary, but the College cannot do impossibilities. Rome, as Miss Yapp observes, was not built in a day.

Nurses do not want trade unions, but they do desire to be protected against the avarice of employers who are deliberately using the present superfluity of supply to advance their own interests.

While protesting against this clause in Miss Yapp's letter, I think she is to be congratulated upon her spirited defence of the rights of her professional sisters.

DAGMAR.

